# Identification, Analysis and Characterization of Base Units of Bird Vocal Communication: The White Spectacled Bulbul (*Pycnonotus xanthopygos*) as a Case Study

**DOI:** 10.3389/fnbeh.2021.812939

**Published:** 2022-02-14

**Authors:** Aya Marck, Yoni Vortman, Oren Kolodny, Yizhar Lavner

**Affiliations:** ^1^The Department of Ecology, Evolution and Behavior, The Hebrew University of Jerusalem, Jerusalem, Israel; ^2^Department of Animal Sciences, Hula Research Center, Tel-Hai College, Tel-Hai, Israel; ^3^Department of Computer Science, Tel-Hai College, Tel-Hai, Israel

**Keywords:** repertoire, bird call detection, bird vocalization, vocal units, White Spectacled Bulbul, *Pycnonotus xanthopygos*, deep learning, unsupervised learning

## Abstract

Animal vocal communication is a broad and multi-disciplinary field of research. Studying various aspects of communication can provide key elements for understanding animal behavior, evolution, and cognition. Given the large amount of acoustic data accumulated from automated recorders, for which manual annotation and analysis is impractical, there is a growing need to develop algorithms and automatic methods for analyzing and identifying animal sounds. In this study we developed an automatic detection and analysis system based on audio signal processing algorithms and deep learning that is capable of processing and analyzing large volumes of data without human bias. We selected the White Spectacled Bulbul (*Pycnonotus xanthopygos*) as our bird model because it has a complex vocal communication system with a large repertoire which is used by both sexes, year-round. It is a common, widespread passerine in Israel, which is relatively easy to locate and record in a broad range of habitats. Like many passerines, the Bulbul’s vocal communication consists of two primary hierarchies of utterances, *syllables* and *words*. To extract each of these units’ characteristics, the fundamental frequency contour was modeled using a low degree Legendre polynomial, enabling it to capture the different patterns of variation from different vocalizations, so that each pattern could be effectively expressed using very few coefficients. In addition, a mel-spectrogram was computed for each unit, and several features were extracted both in the time-domain (e.g., zero-crossing rate and energy) and frequency-domain (e.g., spectral centroid and spectral flatness). We applied both linear and non-linear dimensionality reduction algorithms on feature vectors and validated the findings that were obtained manually, namely by listening and examining the spectrograms visually. Using these algorithms, we show that the Bulbul has a complex vocabulary of more than 30 words, that there are multiple syllables that are combined in different words, and that a particular syllable can appear in several words. Using our system, researchers will be able to analyze hundreds of hours of audio recordings, to obtain objective evaluation of repertoires, and to identify different vocal units and distinguish between them, thus gaining a broad perspective on bird vocal communication.

## Introduction

Vocal communication is an essential tool for transferring information. It serves a diverse range of species and is a topic of multi-disciplinary interest. Studying the regularities and contexts of bird vocalizations may provide keys to understanding numerous aspects of bird behavior. While being an essential part of various species’ biology, the study of vocal attributes and the inference of the signaling properties remains a major challenge. This is because the information conveyed by vocal communication includes many components and facets that include physical attributes such as amplitude, frequency, rhythm, and intensity, as well as more complex aspects such as syllables, words, phrases and more (Kershenbaum et al., 2016). In addition, audio recordings produce a vast amount of digital data per vocalization. Furthermore, these parameters may be expressed differently, which leads to different patterns and correlations between populations and individuals that can be difficult to identify and even predict. This raises intriguing questions about the meaning of animal sounds (Bruno and Tchernichovski, 2019).

According to a study of blue tits, for example, there is a correlation between the call length in males’ courtship songs and extrapair paternity. In this case, the call length provides information about the quality of the singer (Kempenaers et al., 1997). Similar patterns were found with respect to rhythm in sparrows (*Passerculus sandwichensis*) and to variability of calls in warblers (*Sylvia communis*) (Balsby, 2000; Sung and Handford, 2020). From the calls’ characteristics we can reveal information not only at the level of the individual, but also at the level of the species. For example, studies have shown that species with a large repertoire typically have plastic and non-permanent songs, indicative of learning abilities throughout their lifetime in the vocal domain. These species are called *open-ended learners* (Robinson et al., 2019). Such large repertoires introduce multiple challenges when aiming to unravel the signaling properties behind the vocalizations. For instance, large repertoires have been found to indicate a high reproductive success in some species (Robinson and Creanza, 2019), yet, in Botero et al. (2009), it was demonstrated in tropical mockingbird (*Mimus gilvus*) that the variation between vocal expressions decreased as the bird aged, and the expressions became more consistent. In this study system, individuals with more consistent performance tended to achieve higher dominance status and greater reproductive success (Botero et al., 2009). These unexpected patterns demonstrate the diversity and complexity of vocal communication systems.

Manual annotation and analysis of bird song is laborious, time-consuming, and prone to subjective bias. Deep learning and algorithms for extracting audio parameters have the potential to overcome these limitations and challenges of reproducibility and of scaling up to large datasets. In recent years, analyzing digital recordings has benefited from the development of reliable automatic algorithms and deep learning, such as available software for syllable recognition and clustering (DeepSqueak, Coffey et al., 2019), an online tool for bird species identification (BirdNET, Kahl et al., 2021) and robust software for animal call detection in large and noisy databases (ORCA-SPOT, Bergler et al., 2019). Still, in many cases researchers rely on subjective naming of calls and on manual division of vocal units. In addition, in many studies the manual analysis is based on a limited amount of data and may miss out patterns which may be revealed only if enough data is automatically processed and analyzed. In a broader scope, the development of advanced automated tools for bio-acoustic analysis can support large-scale research and reveal organisms’ vocal communication patterns, may facilitate monitoring of populations, and can be leveraged for management and conservation efforts in natural environments (Righini and Pavan, 2020; Kahl et al., 2021).

In this study, using automatic signal processing algorithms and deep learning, we analyzed White Spectacled Bulbul (Pycnonotus xanthopygos) vocalizations. This species is a common, widespread passerine, and was selected as our model since it is characterized by tight social bonds between individuals and a wide repertoire of vocalizations (Shirihai and Svensson, 2018), used year-round by both sexes. We analyzed and characterized 660 base units of the White Spectacled Bulbul from recordings of 14.5 h, to investigate its repertoire and its use of different vocal units. Our analyses show that Bulbul calls are complex vocalizations—*words*, most of them composed of more than one base unit—*syllable*. The complexity of the Bulbuls’ vocal communication can be revealed by intuitive hearing as well as by inspecting spectrograms, or by a more elaborate analysis. However, here we present a set of quantitative automatic methods that make up a pipeline of automatic detection of Bulbul calls, and an analysis of these vocal units that allows classification into different groups, both by supervised and unsupervised learning. These methods (1) allow objective validation of the robustness of words’ and syllables’ classifications; (2) carry out automatic identification and classification to pre-defined classes; and (3) provide the basis for a fully automated process of defining the word and syllable repertoires of a species or an individual.

Our analyses show that the same syllables are used in different words and in distinct geographic populations. This pattern is very likely to indicate a complex hierarchical structure (Kershenbaum et al., 2016) and that the White Spectacled Bulbul is an open-ended learner vocalizing species. Furthermore, this pattern can imply the existence of a more complicated form of communication. The hierarchy of syllables and words provides a basis for investigating syntax questions that are today the focus of widespread interest (Menyhart et al., 2015; Suzuki et al., 2016; Bruno and Tchernichovski, 2019; Searcy and Nowicki, 2019).

## Materials and Methods

**FIGURE 1:**
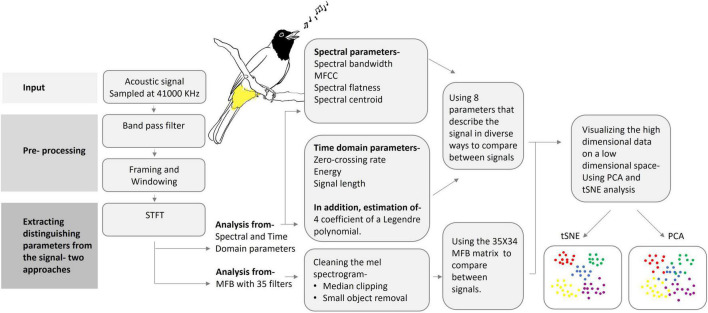
A schematic block diagram of the audio analysis process: from pre-processing to data reduction and visualization. (Artwork by Aya Marck).

A block diagram of the processing and analysis stages of different base units of Bulbul’s vocalization is depicted in Figure 1. Each of the procedures used in each block is detailed below.

### Data Set

**FIGURE 2:**
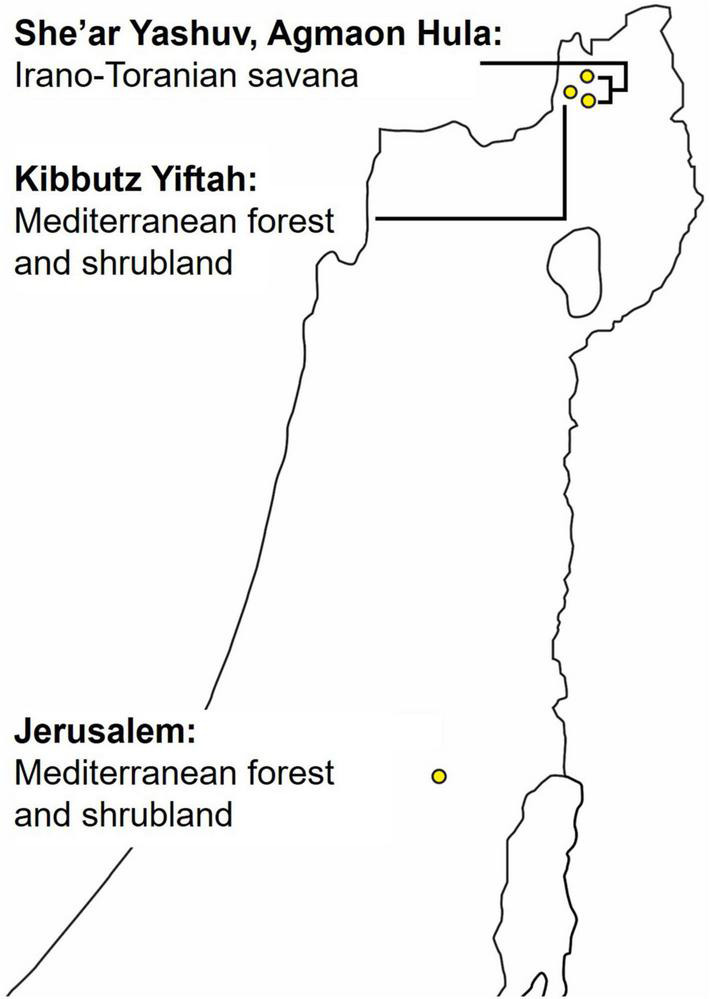
Map of recorders’ locations: three locations in the Hula valley and one in Jerusalem.

**FIGURE 3:**
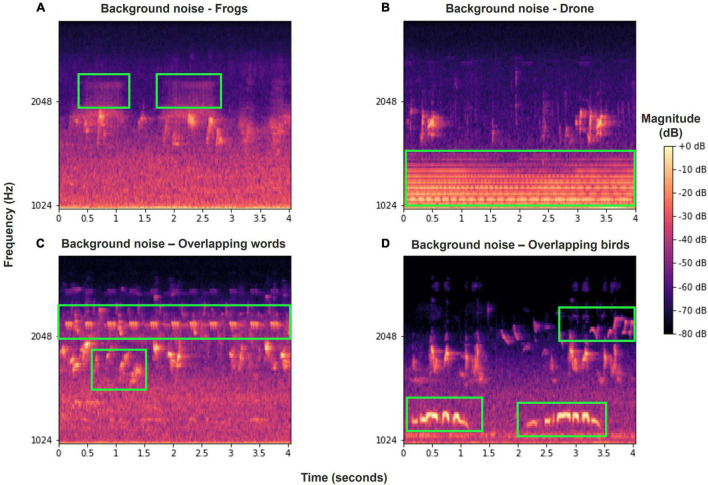
Various examples of background noise or overlapping Bulbul calls in our recordings, marked in green frames. **(A)** Frog vocalizations; **(B)** drone sound; **(C)** overlapping words of other Bulbuls and other birds; **(D)** overlapping calls of other birds. Most of these noise events are filtered out using our pre-processing methods.

Our dataset was collected from eight SM4 automatic recorders of Wildlife Acoustics (Wildlife Acoustics, 2018) placed at four different locations (Figure 2), two recorders at each. The two recorders were placed several hundreds of meters apart to ensure that more than one individual was recorded in each location. The recordings were taken for a period of 6 months to a year, at dawn, noon, and dusk, for a total of 4 h per day, with each recording lasting from 30 to 60 min. Overall, more than 7,000 h were recorded. Six of the recorders were located in northern Israel—four in the Hula valley (She’ar Yashuv and Agmon Hula) and two in a nearby location on the Naftali Mountain range (Yiftah) that is characterized by different habitat and weather. The last two recorders were installed in the bird observatory in Jerusalem, a distinct population for comparison. Since all the recordings were carried out in natural habitats, they contain many types of background noise including other birds and animals, weather sounds and artificial sounds. We used several methods (bandpass filtering, median clipping, and small object removal) described in section “Word Analysis” to filter out the different noises (Figure 3).

### Pre-processing

The acoustic signal was sampled at 44,100 KHz and filtered using a Band-Pass filter between 1 KHz and 3.5 KHz to eliminate background noise and preserve frequencies relevant to the Bulbul’s vocalization. The signal was divided into short segments, either consecutive segments of equal size (0.5–1 s each, similar to the typical bird vocalization duration) with 50% overlap for automatic detection of acoustic events, or of variable size for extracted words or syllables. In both cases, for each segment the discrete short-time Fourier transform (STFT) is calculated using:


$$X\left(n,\omega_{k}\right)=\sum_{m}x\left(m\right)\cdot w\left(n-m\right)\cdot e^{-i\frac{2\pi }{N}km}$$

where *x*(*n*) is the acoustic signal, *w*(*n*) is a han window used to multiply each frame, where frames of 512 samples (∼12 ms) with a hop size of 128 samples are regularly used. Consequently, a mel spectrogram was computed for each segment. Mel scale is a logarithmic-like scale based on the human auditory system that represents the sound frequencies in a similar way to how we and other animals perceive.

### Acoustic Feature Analysis for Analyzing Syllables

In order to compare between vocalization units, we extract several parameters from each signal. Features were extracted both in the time-domain (e.g., zero-crossing rate and energy) and frequency-domain (e.g., spectral centroid and spectral flatness, MFC coefficients). The following features were used to characterize the spectrum:

(a) **Spectral Centroid**(*S*_*c*_) measures the center of mass of the spectrum, and is calculated as:

$$S_{c}=\frac{\sum_{k=0}^{N-1}f\left(k\right)\left|X\left(k\right)\right|}{\sum_{k=0}^{N-1}\left|X\left(k\right)\right|}$$

where | X(k)| is the magnitude of the kth’ bin of DFT, and f(k) is its center frequency.

(b) **Spectral Flatness** (*S*_*f*_) measures how tone-like (*S*_*f*_ ≈ 0) or noise-like (*S*_*f*_ ≈ 1)) is the sound and is computed as the ratio of the geometric mean to the arithmetic mean of the energy spectrum:

$$S_{f}=\frac{\sqrt[{N}]{\pi_{k=0}^{N-1}\left|X\left(k\right)\right|}}{\frac{1}{N}\sum_{k=0}^{N-1}\left|X\left(k\right)\right|}$$

(c) **Spectral Bandwidth**, as defined in Klapuri and Davy (2007), which is a weighted standard deviation of the spectrum in a given audio segment:

$$B={\left(\sum_{k=0}^{N-1}\left|X\left(k\right)\right|{\left(f\left(k\right)-f_{c}\right)}^{2}\right)}^{1/2}$$

(d) **MFB** (log mel filter bank) are a set of filters arranged according to a mel-scale, a perceptually based frequency scale that aims to mimic the frequency perception of the human auditory system (Davis and Mermelstein, 1980). MFB is widely used in audio signal processing including bird analysis and music signal processing. It is calculated by using Discrete Fourier transform of each frame and applying overlapping triangular filter banks, where each filter output is a weighted sum of magnitudes of frequency bins within its support. Data reduction is also a benefit of this computation.
(e) **Mel Frequency Cepstral Coefficients (MFCC)** The MFCCs, derived from the MFB by applying a discrete cosine transform are very common features in audio analysis. A total of 13 coefficients are computed for each frame, where the first four are used for the analysis.
  In Addition, two time-domain parameters were computed:
(f) **Zero Crossing Rate (ZCR)** is defined as the number of times an audio waveform changes its sign within the duration of the signal, and is calculated as:

$$ZCR=\frac{1}{2}\cdot \sum_{n=1}^{K-1}\left|sign\left(x\left(n\right)\right)-sign\left(x\left(n-1\right)\right)\right|$$

Where K is the signal length.

(g) **Fundamental frequency** *f*_0_− which is evaluated using the YIN (De Cheveigné and Kawahara, 2002) or the PYIN (Mauch and Dixon, 2014) algorithms.

#### Legendre Polynomials

In many passerine species, most of the spectral energy of the vocalization is concentrated around the fundamental frequency (Nowicki, 1987; Podos, 2001) since the avian vocal tract attenuates a greater part of the energy of higher harmonics. It is therefore reasonable to assume that a considerable portion of the information conveyed by bird vocalization may be attributed to the intonation, i.e., the fundamental frequency contour.

In order to extract this information quantitatively, we modeled the fundamental frequency contour using a low degree Legendre polynomial, enabling it to capture the different patterns of variation from different vocalizations, so that each pattern could be effectively expressed using only 3–4 coefficients.

This analysis may help us characterize and visualize the fundamental frequency patterns of various syllables which were subjectively divided to different groups.

The usage of the Legendre polynomials for modeling the fundamental frequency was employed in different applications of speech and audio. For example, it has been used to model pitch contour for synthesizing intonation (Zhou et al., 1984), to describe mathematically the nuclear accents found in English in the English isles and to use it for intonation labeling (Grabe et al., 2007). It was also used for automatic language identification (Lin and Wang, 2005), as well as to detect sarcasm in speech and for analyzing prosody (Rakov and Rosenberg, 2013; Rakov, 2019).

The Legendre polynomials is a system of orthogonal polynomials defined as:

$P_{n}\left(t\right)=\frac{1}{2^{n}n!}\frac{d}{dx^{n}}{\left(t^{2}-1\right)}^{n}$ (Rodrigues formula), where *P*_*n*_(*t*) is the n-th order term. It can also be expanded with the polynomials {1, *t*, *t*^2^ … } using Gram-Schmidt process.

According to this definition the first four terms are:


$$P_{0}=1,P_{1}=t,P_{2}=\frac{1}{2}\left(3t^{2}-1\right),P_{3}=\frac{1}{2}\left(5t^{3}-3t\right)$$

Following Grabe et al. (2007), we used the first four polynomials, *L*_0_, *L*_1_, *L_2_* and *L_3_* which represent the average of the signal, its slope, quadratic trend, and wavelike shape, respectively.

The following steps were carried out to fit the Legendre series *p*(*t*) to ${\hat{F}}_{0}\left(t\right)$:

a. A single syllable or vocalization unit is demarcated and excerpted from the acoustic recording. This was carried out using Audacity (Audacity, 2021).
b. The sampled signal *s*(*t*_*n*_), *n* = 0, 1, …, *M*−1 of length M, where *t_n_ = nT_s_* are the time samples, is filtered using a bandpass filter between 700 and 3,900 Hz, based on the range of frequencies characteristic of the White Spectacled Bulbul.
c. For each sampled syllable or vocalization unit the fundamental frequency contour *F*_0_(*t*_*n*_) is estimated with either PYIN (Mauch and Dixon, 2014) or the YIN (De Cheveigné and Kawahara, 2002) algorithms, or by using a simple zero-crossing rate analysis signal *z*(*t*_*n*_). In many cases the latter is preferred, since the pitch detector algorithms (YIN and PYIN) which were developed mainly for speech and music signals, may not be robust enough for noisy bioacoustic data. Furthermore, the ZCR computation yields a good estimation of *F*_0_(*t*_*n*_).
d. A polynomial fit is used after scaling the time axis to be between −1 and 1. The estimated contour, ${\hat{F}}_{0}\left(t_{n}\right)$, is modeled using an m-th degree Legendre series defined as:

$$p\left(t\right)=a_{0}+a_{1}L_{1}\left(t\right)+a_{2}L_{2}\left(t\right)+\ldots +a_{m}L_{m}\left(t\right)$$

where *L*_*j*_(*t*)is a Legendre polynomial and *a_j_* is its corresponding coefficient. The polynomial series is a least square fit to the data ${\hat{F}}_{0}\left(t_{n}\right)$, where the fitting process is carried out by solving an overdetermined set of linear equations of the form:


$$V\left(t\right)\cdot a=w\cdot {\hat{F}}_{0}\left(t_{n}\right)$$

where *V*(*t*) is the pseudo Vandermonde matrix of t, *a* is the vector of coefficients.

**FIGURE 4:**
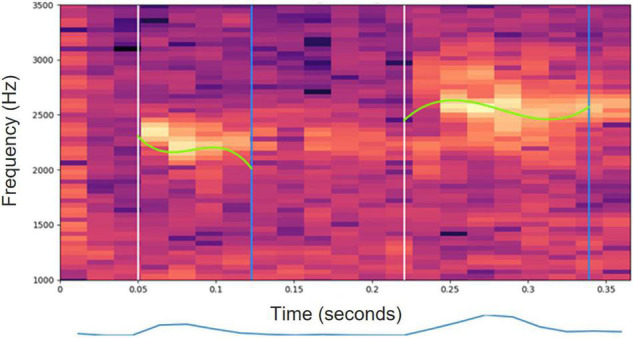
A spectrogram of two syllables of a White-Spectacled Bulbul (*Pycnonotus xanthopygos*). The fundamental frequency contour modeled by a 3rd degree Legendre polynomial is superimposed on the spectrogram. The lower graph depicts the short-time energy of the syllables.

**FIGURE 5:**
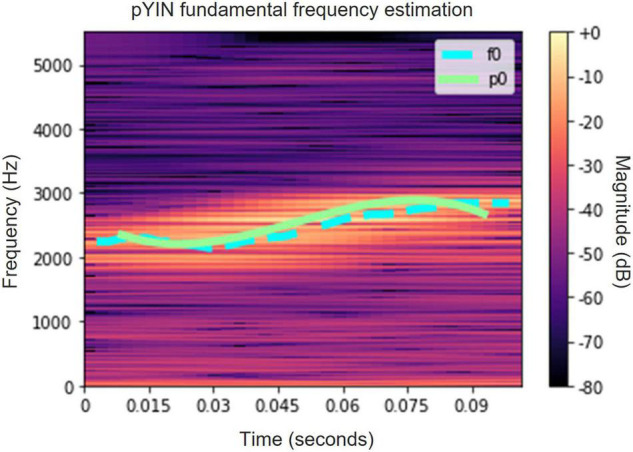
A spectrogram of a single syllable of a White-Spectacled Bulbul (*Pycnonotus xanthopygos*). The fundamental frequency contour is estimated by PYIN (cyan) and by ZCR (light green).

Figures 4, 5 present spectrograms of a word (Figure 4) and of a single syllable (Figure 5), with the fitted Legendre series superimposed.

### Word Analysis

Bulbuls have complex vocalizations which are described as words that consist of several base units—syllables, and intervals in between. Extraction of features from these complex units is complicated due to their various number and type of syllables, as well as their varying intervals. Therefore, we aimed to create one feature vector that describe the entire vocalization. For this, we used the mel-spectrogram, applied to the raw isolated word signal, with 35 mel filters between a low frequency *f_L_* and a high frequency *f_H_*. We set *f*_*L*_to 700 Hz and *f_H_* to 3,900 Hz, according to the range of frequencies for most Bulbul vocalizations.

Consequently, a variation of median clipping (Lasseck, 2014; Fukuzawa et al., 2016) following by a small object removal is applied. These two simple image processing techniques are applied to increase the SNR, since in most of the recordings a high background noise is present:

(1) **Median clipping**—in this technique a binary mask is generated for masking background noise, where for each time-frequency point (*i*, *j*), its corresponding spectrogram value *S*(*i*, *j*) is compared to a threshold value which is based on the median of the corresponding row and column of that point. Thus, the median clipped spectrogram *S*_*mc*_(*i*, *j*)is obtained by:

$$S_{mc}\left(i,j\right)=\left\{ \begin{array}{cc} S\left(i,j\right) & ifS\left(i,j\right)>F\cdot \text{max}(med\left(S_{i}\right),med\left(S_{j}\right)\\ S_{L} & otherwise \end{array}\right.$$

where *F* is a multiplication factor set here to be 3.5 and *S_L_* is the lowest value in the spectrogram which is set to −80 dB.

(2) **Small object removal**—used to remove small blobs which are probably irrelevant to bird vocalizations and may stem from background noise. This is carried out by converting the median clipped mel-spectrogram to a binary matrix, and for each non-zero entry calculating its immediate non-zero neighbors. Non-zero entries whose number of neighbors is below a pre-defined threshold are zeroed, and a binary mask is obtained. The final spectrogram is then obtained by:

$$S_{sor}\left(i,j\right)=S_{mc}\cdot M_{neigh}$$

**FIGURE 6:**
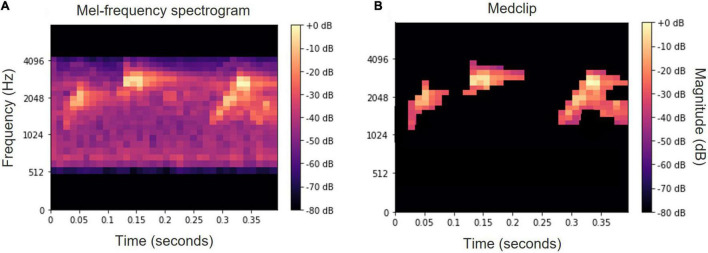
Mel-spectrogram of the word “*tu ti tuyu*” before **(A)** and after **(B)** median clipping and small object removal.

Alternatively, the usage of a white top-hat transform (Sonka et al., 2014) was examined with no significant difference. An example of a mel-spectrogram before and after these processing operations is depicted in Figure 6.

Finally, to compare between feature vectors that represent different words with variable length, we transform the vectors’ dimensions to a fixed size by zero padding. Alternatively, the spectrogram was calculated using a fixed number of frames with fixed duration and variable hop length.

### Synthesis

To demonstrate that Legendre polynomial coefficients can extract most of the vocal information from Bulbul calls (at least as perceived by a human), and to use another method to validate the parameters we use, we generated a synthetic vocalization based solely on these coefficients. The synthesis is carried out using the following steps:

a. For each input signal with one syllable—a pre-processing is applied, which includes downsampling to a sampling rate of 11,025 Hz, bandpass filtering of the signal using cut-off frequencies (700, 3,900) and demarcation of the syllable boundaries.
b. Fundamental frequency contour of the syllable is evaluated, using either pitch detection algorithm or applying a ZCR analysis on the bandpassed signal. The evaluation is carried out using a frame length of 64 samples (5.8 ms) and a step size of 32 samples (2.9 ms). The result of this stage is a vector of consecutive fundamental frequency values.
c. A three-degree Legendre polynomial is fitted to the fundamental frequency contour, and four Legendre coefficients are obtained.
d. Using the coefficients, a Legendre series is fitted for the time points for which the fundamental frequency contour was evaluated.
e. For each frame *m*, a short sinusoid is produced, with frequency *p*_0_(*t*_*m*_) using:

$$x\left(n\right)=Re\left\{A\cdot e^{j\left(2\cdot \pi \cdot p_{0}\left(t_{m}\right)\cdot n+\varphi \right)}\right\}$$

where A is the signal amplitude, n is the sample index, and φ is the phase shift which is corrected for each frame to avoid phase discontinuities. The initial phase is set to φ_*t*_*m*__ = 0 and then for next frames it is set according to the final phase of the former sinusoid:


$$\varphi_{t_{m+1}}\left(0\right)=\left\{ \begin{array}{cc} acos\left(x_{t_{m}}\left(N-1\right)\right)+2\cdot \pi \cdot \frac{T_{s}}{T_{0}\left(t_{m}\right)} & \\ if0\leq \varphi_{t_{m}}\left(N-1\right)<\pi & \\ 2\cdot \pi -acos\left(x_{t_{m}}\left(N-1\right)\right)+2\cdot \pi \cdot \frac{T_{s}}{T_{0}\left(t_{m}\right)} & \\ if\pi \leq \varphi_{t_{m}}\left(N-1\right)<2\pi & \end{array}\right.$$

where *N* is the frame size, *T*_0_(*t*_*m*_) is the fundamental period evaluated for the *m*_th_ frame and *T_s_* is the sampling interval.

The concatenation of all the frames yields a chirp-like signal, with a fundamental frequency contour according to the evaluated Legendre series.

### Visualizing by Data Reduction

To validate the division into different syllables or words made by listening and observation, which may be subjective, we first tagged 660 vocalizations, containing 1,004 syllables, all collected from nine separate audio files that were recorded at the same location with the same device, each lasting an hour. Further, an algorithm for dimension reduction was utilized, based on the spectral analysis data, to objectively examine the proposed grouping, both for words and syllables. We used PCA (Principal Component Analysis) and t-SNE algorithm (t-distribution Stochastic Neighbor Embedding, Van der Maaten and Hinton, 2008) for dimension reduction and visualization. Both methods perform a mapping from a high dimension to a low dimension of 2–3, so that the proximity or distance between points in the high dimension is maintained in the low dimension. Syllables feature vectors were reduced from 8 dimensions to 2, and words feature vectors from 1,190 dimensions to 2. We expect that syllables or words that were divided by listening into one group should be in the same cluster, whereas feature vectors from sounds classified subjectively as belonging to different groups would be divided by the algorithm into different clusters and would be far apart. While methods based on linear algorithms such as PCA may not yield clear results, the usage of a nonlinear method such as t-SNE, may show the clustering and separation of sounds in a manner similar to their definition on an auditory basis.

For analysis and computations, we used Python 3.8 and suitable packages; Librosa (McFee et al., 2015) for audio signal processing, and Scikit-learn (Kramer, 2016) for data analysis and data reduction. Dataset for this analysis and codes are available on GitHub (see Data Availability Statement).

### Detection of Bulbul Calls

The analysis of words and syllables receives as input an audio signal where the relevant vocalization is located. It is therefore necessary to identify and extract the desired call events from long and noisy recordings. This can be done manually; however, the number of call events that can be derived in this way is limited. A machine learning approach should be applied in order to extract thousands of vocalization units for further analysis. We used several Deep Neural Networks (DNN), and in particular Convolutional Neural Networks (CNN) (Goodfellow et al., 2016) to automatically detect the Bulbuls’ calls in the recordings. Most of the recordings are between half an hour to 1 h long and contain intense background noise as well as other birds’ and other animals’ vocalizations (including human speech). Several models were tested for the detection:

(a) A CNN with 5 blocks of convolution and max pooling layers, connected to a 90 hidden units fully connected (FCN) layer and an output layer with a total of 1,117,781 trainable parameters;
(b) A resnet architecture with 14 convolution layers in resnet blocks, connected to a FCN layer of 90 hidden units with a total of 625,397 trainable parameters;
(c) A mini-Xception model (Chollet, 2017, 2021) with 7 convolution layers and a total of 717,249 trainable parameters.

The input for all the models was obtained by dividing the acoustic signal into consecutive segments of 1 s each, with an overlap of 50%. For each segment, a log mel-spectrogram was calculated by using frames of 2,048 samples (∼48 ms for fs = 44,100) and hop size of 700 samples. The mel-spectrogram is a matrix of 50 × 60 (number of mel filters x number of time bins), which was pre-processed by median clipping and small object removal for noise reduction.

**FIGURE 7:**
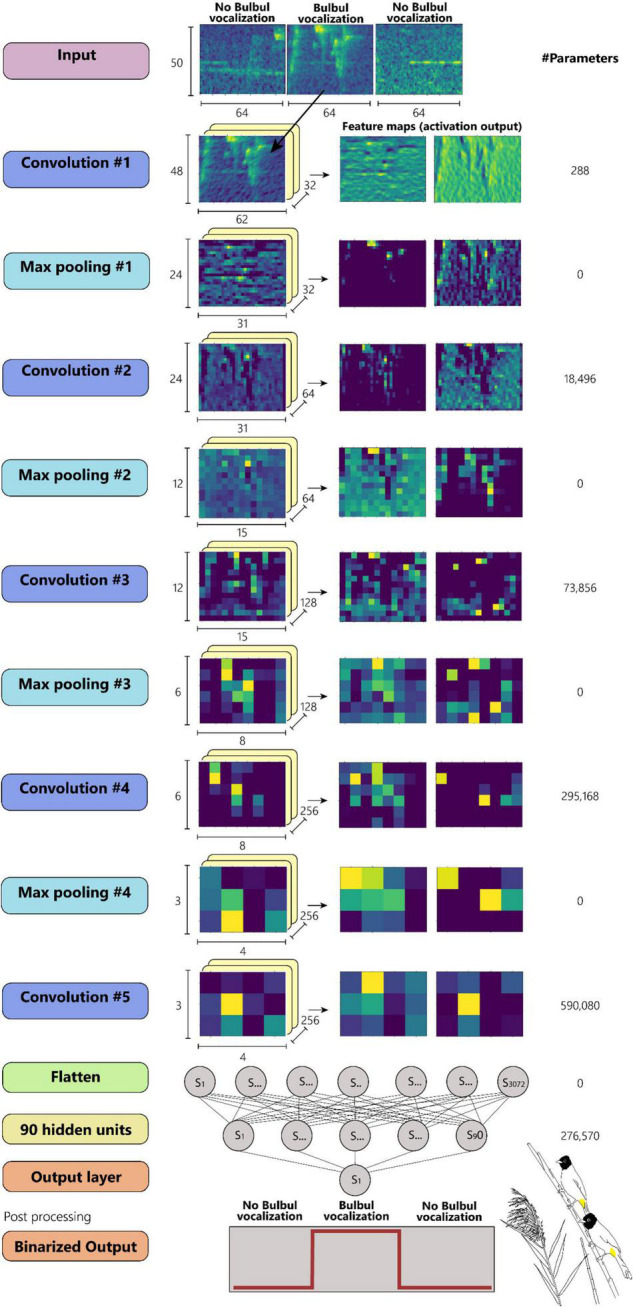
A CNN model configuration with 5 convolutional layers. (Artwork by Aya Marck).

The CNN model (Figure 7) is composed of 5 blocks of convolution: a first block of a convolution layer with 32 3 × 3 kernels following by a max-pooling layer (2 × 2) and a Relu activation function. The following convolution blocks are the same, where the number of kernels doubles at each block. After flattening the output feature map of the final convolution layer, a fully connected layer of 90 units is applied with dropout of 0.5. Finally, the output layer with one unit and a Sigmoid activation function and threshold value of 0.5 yields a binary output of (Bulbul = 1/non-Bulbul = 0). Consecutive segments predicted as Bulbul (1), were merged into one call event for further processing.

The training set for the detection included 57 recordings of variable durations, with a total duration of more than 8 h, which were annotated manually. The annotations were made by examining the spectrograms and listening to the corresponding sounds, and the start and end times of each identified vocalization were listed (“strong labeling,” Mesaros et al., 2021). This dataset contains several thousands of Bulbul calls, along with other birds, human activity, and many other sounds from various sources. We used 70% of this dataset for training and the remainder for testing. Out of the training data, 10% was randomly selected and served for validation. A segment-based evaluation is applied, and each segment is considered a bird call if at least 30% of it is overlapped with an annotated Bulbul vocalization event.

For data augmentation we used five different methods to increase variability of the data, thus improving the robustness of the networks: (a) Adding white noise to each Mel-spectrogram. (b) Applying horizontal random flip to the mel- spectrogram. (c) Applying a random zoom-in with factors between (−0.2 to −0.01). (d) Time stretching or compressing in the acoustic signal in the range (0.7–1.3). (e) Pitch shift of the signal in the range (0.94–1.06). This process tripled the size of the input data. Dataset for this network and codes are available on GitHub (see Data Availability Statement).

## Main Results

### Convnet Network Performance—Bulbul Event Detection

We measured the performance of the DNNs in detecting Bulbul’s vocalizations using a test dataset of 3 h with several thousand individual calls, which also contained high background noise from other birds and animals, as well as anthropophonic and geophonic sounds (Righini and Pavan, 2020). The test dataset was pre-processed with the same procedure used for the training dataset, which included MFB calculation, median clipping, and small object removal. The test set was randomly selected from the recordings dataset, and a segment-based evaluation was carried out using a 1 s segment.

A correct identification rate of 75% (True Positive Rate, or recall, i.e., the ratio between the number of Bulbul vocalization segments correctly identified, to the total number of segments with Bulbul vocalization in the test recording set) was yielded by the CNN described in section “Detection of Bulbul Calls,” with a relatively low False Positive occurrences of less than one third (27%) of the True detections. In a manual examination of the results, the non-identified calls (false negatives) were usually further from the microphone or very noisy. The Resnet and the mini-exception models yielded similar results.

### A Wide Repertoire of Distinct Words That Repeat Themselves

**FIGURE 8:**
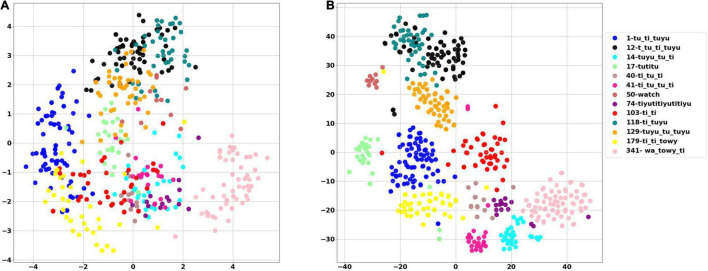
The PCA score plot of the first two PCs of the word analysis **(A)**, and tSNE analysis **(B)** where each color represents one unique naming tag. Instances of each word are grouped together by both unsupervised algorithms. For example, all of the pale pink points are grouped together, as they all represent word number 314—”*wa towy ti.*”

The White Spectacled Bulbul demonstrated a broad vocabulary of more than 30 distinct words. Over 660 calls were tagged, named, and analyzed, and were manually categorized as 13 different words (see examples in Supplementary Figure 1). Each word was represented by a mel spectrogram of 35 × 34 which was cleaned and filtered as described in section “Word Analysis.” Two computational analyses were performed to visualize the 1,190 dimensions of the mel-spectrogram as a two-dimensional map—PCA and t-SNE. Figure 8A shows the PCA result where each dot represents a word, and each color represents one unique naming tag. As shown, the vocalizations that were perceived and categorized as belonging to the same word by a human expert were also mapped to the same region on the 2-D plane of the unsupervised PCA (groups of similar colors). This grouping is further demonstrated using a second unsupervised method: the tSNE analysis (Figure 8B). The tSNE plot places most of the words (different colors) in well-defined, separate clusters. These results suggest that Bulbuls use distinct words that appear non-random as they repeat themselves across different recordings throughout the year. Our manual process of naming words and categorization aligns well with these unsupervised dimensionality reduction analyses.

### Different Words Are Composed From the Same Shared Base Units

**FIGURE 9:**
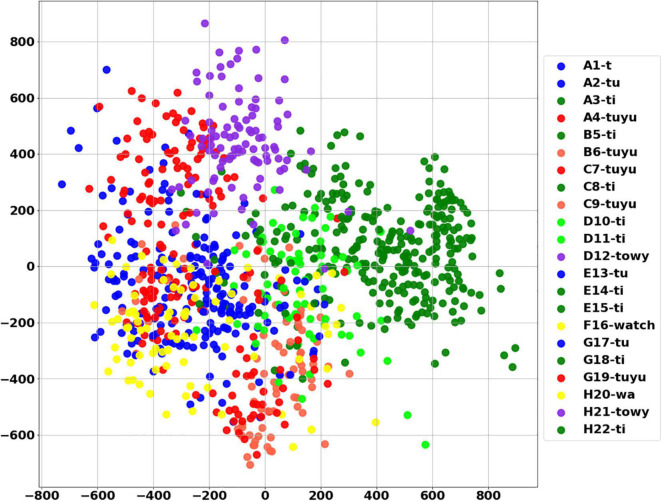
Visualization of syllables using PCA. Explained variance ratio (first two components): (0.43169461 0.2822671).

**FIGURE 10:**
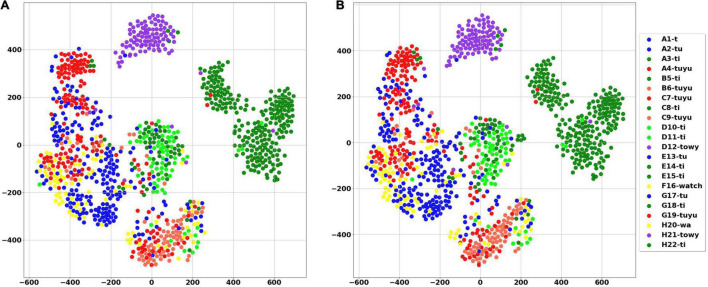
Visualization of syllables using tSNE. Each syllable is represented by a single point. **(A)** The analysis includes all parameters. **(B)** The analysis includes only 4 Legendre coefficients. In both figures same syllables appears in different words. For example, the purple dots that represent the syllable “*towy*,” were excerpted from two different words (denoted by the letters D and H) and are clustered together. Similarly, the syllable “*ti*” (dark green), derived from six different words, resides in the same region both in PCA and tSNE analyses.

A total of 1004 audio signals containing 22 different syllables were excerpted from the words and manually categorized and tagged with a number by listening and examining the spectrogram (see examples in Supplementary Figure 2). Syllables were represented by an eight-parameter feature vector, which includes—syllable length, spectral flatness, spectral centroid, bandwidth, and four Legendre polynomials coefficients—based on the fundamental frequency contour. The results of both PCA and tSNE are provided in Figures 9, 10A, respectively. Figure 10B shows that using only the Legendre coefficients as parameters is sufficient to describe the variance of the acoustic signal. In Figure 10A, words (denoted with capital letters) are composed of different syllables. The same syllables often appear in different words. This analysis can serve as an effective test or validation for manual assessments; for example, we found that two syllables from different words that clustered together were initially misidentified as different syllables. Later listening and visual inspection of the spectrograms confirmed that they represent a single syllable in different contexts. These results show that there is a collection of distinct syllables that repeat themselves and appear in different words, indicating that different words are constructed from the same shared units of similar and non-random syllables.

### Classification of Words From a New Dataset

The final stage in the automatic pipeline presented above is to classify the segments detected as Bulbul vocalizations by the deep CNN into their corresponding classes. For this purpose, we first applied the trained CNN model presented in section “Detection of Bulbul Calls” to a 3-h long recording dataset. A group of 800 segments were detected as Bulbul calls and demarcated using the model. These were used to construct two test datasets: 1. A dataset of 126 segments consisting only of words recognized as belonging to the predefined repertoire, 2. A dataset containing 200 segments selected randomly from all detected segments. Later examination found that these included both known words (101 segments) and unknown segments that cannot be classified into existing word-categories. These segments include words that the researcher has yet to annotate, a mix of words (when more than one bird sings in unison), fragments of words (initial or final syllables), as well as a few false positives (i.e., not a Bulbul vocalization).

Using the dimensionality reduced PCA representation described in section “A Wide Repertoire of Distinct Words That Repeat Themselves” for training, the high-dimensional mel-spectrograms of the test segments were projected into the reduced PCA space to produce a low-dimensional representation of the test data. Consequently, three simple classifiers were used to classify the test segments: A k-nearest neighbor (KNN) classifier with K = 3, a nearest centroid classifier, where the prediction of the test word is set according to the label of the closest centroid among all centroids of the training groups, and a Support Vector Machine (SVM) with a radial basis function kernel. The classification results of applying the classifiers on the first fully annotated test set are summarized in Table 1. As can be seen, using a 10-dimensional representation a very high classification accuracy was obtained, of 95.2, 94.4, and 96.8%, for the nearest centroid, KNN and SVM, respectively. Even better scores were achieved using a 100 dimensional representation.

**TABLE 1:** A summary of the classification results with three different classifiers and with a different amount of dimensionality reduction components.

Accuracy % (# errors)			n_components
SVM	KNN	Nearest centroid	
98.4 (2)	97.6 (3)	96.8 (4)	100
96.8 (4)	94.4 (7)	95.2 (6)	10
92.1 (11)	92.8 (9)	92.9 (10)	5
84.9 (19)	85.7 (17)	82.5 (22)	3
70.6 (37)	71.4 (36)	70.6 (37)	2

*The classification accuracy is the ratio between the number of correctly classified words and the total number of words in the test dataset (of 126 words).*

The same pre-processing was used in the second dataset, in which the detected words were selected randomly. However, to reject the unrelated segments detected erroneously as Bulbul, a threshold value was set, based upon the distances of all training word samples from their respective closest centroid. For each test segment, whenever the nearest centroid distance is higher than the threshold, this instance is discarded. Using this procedure, most of the non-Bulbul segments were rejected, as well as some Bulbul vocalizations. A classification accuracy of 77% was achieved for this dataset.

Evidently, when the CNN is used to identify words that were included in the training repertoire, this classification tool can guarantee a fully automated process, with very high recognition rates. When unknown vocalizations are also considered, recognition rates are lower. These can be improved in a number of ways; the researcher may inspect the detected words before classification to remove the irrelevant vocalizations, and high accuracy results could be also achieved by applying simple classification tools.

## Discussion

The field of bio-acoustic research is rapidly expanding, with technological advances facilitating new approaches to fundamental biological questions and new applications in conservation. This includes utilization of deep neural networks in ecological studies for monitoring and processing large datasets of field recordings (Bergler et al., 2019; Dufourq et al., 2021). As in our framework, most of these studies use a convolutional neural network, with an augmentation approach similar to ours. These are typically complemented by pre-processing and post-processing stages, which are tailored to the specific species, environment and future use of the data. Several frameworks that automate the analysis of animal communication and quantify vocal behavior have been developed. These include studies and software packages such as Sound Analysis Pro (Tchernichovski and Mitra, 2004), which includes automatic segmentation and calculation of acoustic features as well as clustering of vocal units, and DeepSqueak (Coffey et al., 2019), in which a regional CNN (Faster rCNN) and k-means are used to detect and cluster mouse ultrasonic vocalizations. Similar to our syllable analysis approach, these programs extract different acoustic features to characterize and differentiate between vocal units, and use unsupervised methods to visualize and classify the data. In our study, however, we used solely Legendre Polynomials to capture the shape of the fundamental frequency contour, demonstrating that very few coefficients are sufficient to effectively express the different patterns of syllable variation. Goffinet et al. (2021) used a variational autoencoder (VAE) to extract features in reduced latent spaces, employed on mouse USV and zebra finch vocalizations, and demonstrated the effectiveness of a latent space representation when compared with handpicked selected features in different vocal analyses. This kind of analysis shares the data-driven approach applied in our word analysis process and seems effective for recognition of patterns and characterization of units from the complex and high-dimensional data of vocal communication. However, most of these studies used recordings in artificial environments (did not contain high background noise) or were designed for a specific species, and it is challenging to apply them to non-model passerine in the field.

Several significant cross-disciplinary challenges in the study of vocal communication still exist (Prat et al., 2017; Teixeira et al., 2019; Mercado and Perazio, 2021). These methodological challenges arise due to the vast amount of digital data produced, the large number of parameters that can be potentially extracted (e.g., frequency, duration, pitch, etc.) and the lack of clear hypotheses regarding the parameters and the signals they convey (Suzuki et al., 2019).

A basic conceptual challenge is the categorization of vocal units, and more generally, the definition of the repertoire. Most often, the construction of a repertoire dictionary is an expression of guidelines defined by the researcher. Vocal units can be categorized, for example, by a “hard” division (only the exact call is considered the same word), or a “soft” division (a variety of calls that sound alike are considered the same word). This scheme may or may not express the way that animals perceive or use their repertoire (Kershenbaum et al., 2016; Mercado and Perazio, 2021). Moreover, while human perceptual properties may be fit for such a task, this may cause additional methodological challenges, may add room for inconsistencies and reduce reproducibility. Thus, quantitative validation of vocal categorization may aid in overcoming these challenges.

By taking advantage of the benefits of automatic analysis we overcome these challenges in two ways:

1. **Processing large amounts of data**—Our CNN model which is used for identifying Bulbul sounds is highly efficient since it reduces manual work and processes big datasets. Further, deep learning has the ability to recognize discriminative patterns in a non-trivial way and can consider combinations of multiple variables that the human auditory system may miss. With the right adjustments, this network can be used with noisy recordings taken in the wild, to identify various bird species and perhaps other animals with wide vocal repertoire. As part of a post-processing, the call events can be analyzed—both for audio analysis (e.g., clustering and comparing between calls) and for statistical analysis (e.g., call events per day and daily or seasonal fluctuation of calls).
2. **Validation and avoidance of biases**—Our automatic analysis for syllables and words can validate our subjective classification and makes it possible to significantly reduce biases that may occur in manual analysis. The categorization process can be done manually by characterizing vocal units in a parametric analysis (like frequency, etc.) into distinct words. But, it is a long and tedious process, made more difficult by large amounts of audio files. Linear and non-linear dimensionality reduction and visualization techniques as well as supervised classification schemes can demonstrate that the manual choices which have been made in early stages are to an extent consistent. Further, it can highlight mistakes and reveal new insight about the categorization. As seen in our results, by using PCA, two syllables from different words which were cataloged differently were found to be the same syllable.

In addition to validating the manual work, the use of our syllable analysis tool enables us to compare similar syllables in different geographic regions in order to identify minor differences. Furthermore, the word analysis tool allows to compare dialect differences between populations, identify which words are unique and which are shared, and investigate if these differences are correlated with geographical distances or genetic differences. During our fieldwork, cameras were placed next to some of the automatic recorders. Further behavioral research can be conducted by analyzing these thousands of short video clips using other deep learning models. Additionally, since females and males are morphologically similar, sex and age of the recorded individuals are unknowns. This information, and possible sex differences in vocalizations can be obtained by combining audio recordings, corresponding video clips and DNA samples (research in progress).

Our study revealed a particularly large vocal repertoire produced by various Bulbul populations. We confirmed through our analysis that the repertoire is derived from a combination of the same basic units which are used to generate new or unique words. This result may be explained in at least two ways. First, is that the Bulbul uses an efficient hierarchical repertoire by maximizing a limited stock of syllables for composing a variety of different words. These signal combinations can be cultural or socially transmitted. Another explanation is that syllables are innate and there may be genetic constraints upon the neural control or physiological mechanisms, limiting the production of syllables, while words, which are made up of syllables, can be invented or learned. The combination of the large repertoire together with a vocal structure of words comprised from syllables may suggest that the Bulbul is an open-ended vocal learning species (Cornez et al., 2017).

Our pipeline provides a robust framework that enables us to process large amounts of data with very little manual intervention, and to classify and validate our findings in an unsupervised analysis. Using the pipeline, raw noisy recording can be processed down to the level of a single word or syllable. This facilitates further analysis and research on Bulbul vocal communication, opening the door to investigation of question such as whether the emergence of novel words characterizes isolated populations, whether different Bulbul calls convey a specific message or information and whether the syllable arrangement into words has certain rules that operate over them. The framework’s code and documentation are available on GitHub in the following link: https://github.com/BulbulNET?tab=repositories. The code can be utilized for study of Bulbul vocalizations as is and can easily be adapted to analysis of vocalizations of other passerines that share similarities with the Bulbuls’ vocalization structure. We would be happy to assist in the incorporation of the code or parts of it into new pipelines that are being developed for such studies with the goal of generating new insights into the complex world of animal acoustic communication.

## Data Availability Statement

The analysis methods and datasets for this study can be found in GitHub, in the following link https://github.com/BulbulNET?tab=repositories.

## Author Contributions

All authors listed have made a substantial, direct, and intellectual contribution to the work, and approved it for publication.

## Conflict of Interest

The authors declare that the research was conducted in the absence of any commercial or financial relationships that could be construed as a potential conflict of interest.

## Publisher’s Note

All claims expressed in this article are solely those of the authors and do not necessarily represent those of their affiliated organizations, or those of the publisher, the editors and the reviewers. Any product that may be evaluated in this article, or claim that may be made by its manufacturer, is not guaranteed or endorsed by the publisher.
