# Supplementary material for: Identification, Analysis and Characterization of Base Units of Bird Vocal Communication: The White Spectacled Bulbul (Pycnonotus xanthopygos) as a Case Study
Source: Front Behav Neurosci. 2022 Feb 14;15:812939. doi: 10.3389/fnbeh.2021.812939 (PMC8884146; doi:10.3389/fnbeh.2021.812939)
Supplement: Supplementary file 1 [file Image_1.pdf]

Supplementary Materials

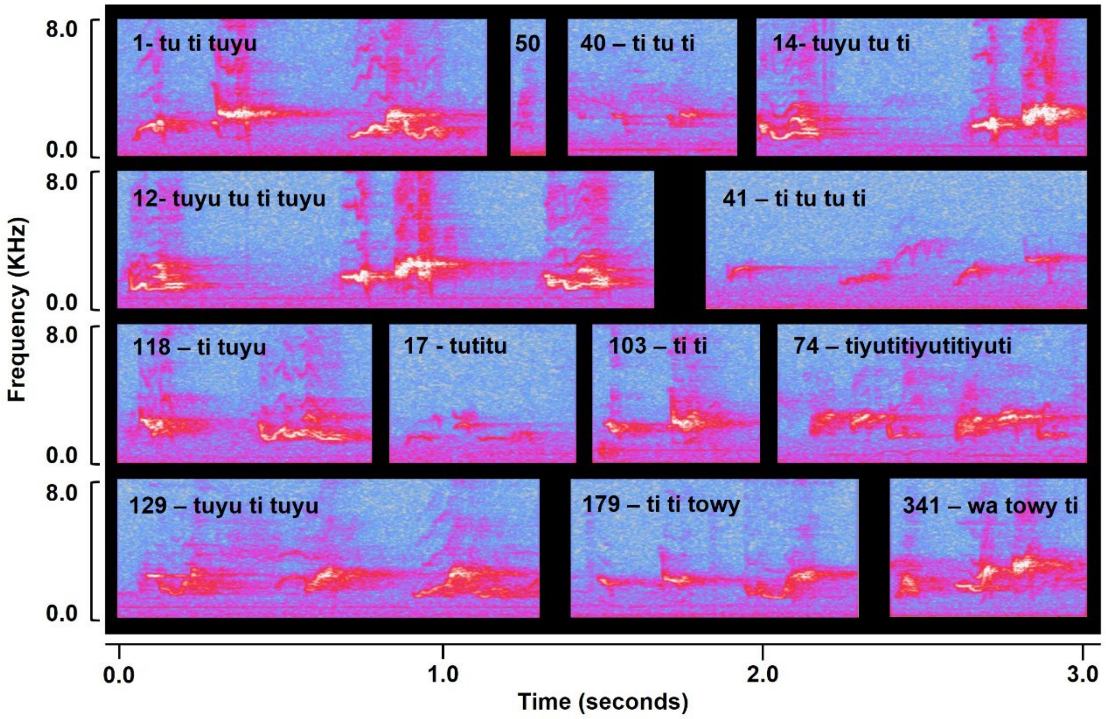

Supplementary Figure S1. Spectrograms of 13 words from the word analysis described in section 3.2 is presented. Numbers and names are presented as they are noted in figure 8.

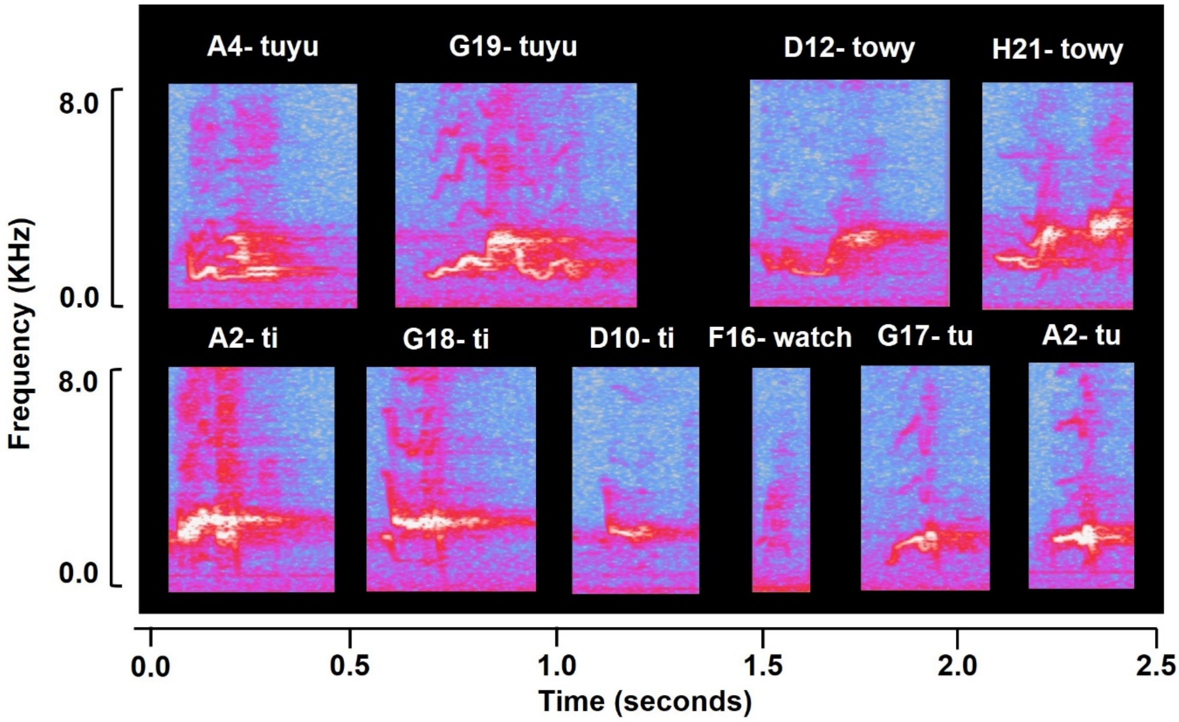

Supplementary Figure S2. Spectrograms of 10 different syllables from the syllable analysis described in section 3.3 is presented. Numbers and names are presented as they are noted in figure 10.
